# Comparisons of biophysical properties and bioactivities of mono-PEGylated endostatin and an endostatin analog

**DOI:** 10.1186/s40880-016-0080-8

**Published:** 2016-01-20

**Authors:** Shan Wang, Yan Fu, Yongzhang Luo

**Affiliations:** The National Engineering Laboratory for Anti-tumor Protein Therapeutics, School of Life Sciences, Tsinghua University, Beijing, 100084 P. R. China; Beijing Key Laboratory for Protein Therapeutics, School of Life Sciences, Tsinghua University, Beijing, 100084 P. R. China; Cancer Biology Laboratory, School of Life Sciences, Tsinghua University, Beijing, 100084 P. R. China

**Keywords:** Endostatin, PEGylation, Antiangiogenic therapy, Drug design, Zinc-binding protein-endostatin

## Abstract

**Background:**

Endostatin (ES) is a well-established potent endogenous antiangiogenic factor. An ES variant, called zinc-binding protein-ES (ZBP-ES), is clinically available; however, its use is limited by rapid renal clearance and short residence time. PEGylation has been exploited to overcome these shortcomings, and mono-PEGylated ES (called M_2_ES) as well as mono-PEGylated ZBP-ES (MZBP-ES) are developed in our study. This study aimed to compare the biophysical properties and biological effects of M_2_ES and MZBP-ES to evaluate their druggability.

**Methods:**

Circular dichroism and tryptophan emission fluorescence were used to monitor the conformational changes of M_2_ES and MZBP-ES. Their resistance to trypsin digestion and guanidinium chloride (GdmCl)-induced unfolding was examined by Coomassie staining and tryptophan emission fluorescence, respectively. The biological effects of M_2_ES and MZBP-ES on endothelial cell migration were evaluated using Transwell migration and wound healing assays, and the uptake of M_2_ES and MZBP-ES in endothelial cells was also compared by Western blotting and immunofluorescence.

**Results:**

Structural analyses revealed that M_2_ES has a more compact tertiary structure than MZBP-ES. Moreover, M_2_ES was more resistant to trypsin digestion and GdmCl-induced unfolding compared with MZBP-ES. In addition, although M_2_ES and MZBP-ES showed comparable levels of inhibiting transwell migration and wound healing of endothelial cells, M_2_ES displayed an increased ability to enter cells compared with MZBP-ES, possibly caused by the enhanced interaction with nucleolin.

**Conclusions:**

M_2_ES has a more compact tertiary structure, is more stable for trypsin digestion and GdmCl-induced unfolding, exhibits increased cellular uptake and shows equivalent inhibitory effects on cell migration relative to MZBP-ES, indicating that M_2_ES is a more promising candidate for anticancer drug development compared with MZBP-ES.

## Background

Angiogenesis, the formation of new capillaries from pre-existing blood vessels, is vital for both physiologic and pathologic conditions. As one of the hallmarks of cancer [1], the process of angiogenesis is exquisitely regulated by a variety of activators and inhibitors [2, 3]. These pro-angiogenic and anti-angiogenic factors constitute a tightly controlled angiogenic balance. Among them, one of the first identified endogenous factors with anti-angiogenic activities was endostatin (ES) [4]. ES, a 22 kD C-terminal fragment of collagen XVIII, can significantly retard tumor growth by targeting endothelial cells in mouse models [5] when assessed by proliferation, migration, tube formation, and numerous signaling pathways. The clinical trials of ES in the United States failed due to insufficient efficacy and problems in the production and formulation [5]. However, mutagenesis of ES by the linkage of MGGSHHHHH to the N terminus stabilizes ES and has made it successfully clinically available [5]. This ES variant, also known as the zinc-binding protein-endostatin (ZBP-ES), was approved by the China Food and Drug Administration (CFDA) to treat non-small cell lung cancer (NSCLC) patients in China in 2005 [6]. As a widely used anticancer drug in China, ZBP-ES has exhibited beneficial therapeutic effects [7–9]. A meta-analysis of clinical data, involving 1953 NSCLC patients, demonstrated that ZBP-ES co-treatment significantly improved the overall response rate and disease control rate of patients who underwent platinum-based doublet chemotherapy by 14.7 and 13.5%, respectively [6]. In another study, the endpoints of progression-free survival (PFS) and overall survival (OS) were evaluated in a total of 110 patients with metastatic melanomas [8]. Significant improvements were observed in favor of the ZBP-ES plus dacarbazine arm compared with the placebo plus dacarbazine arm (PFS, 4.5 vs. 1.5 months; OS, 12.0 vs. 8.0 months). However, hindered by its relatively rapid renal clearance and short circulation half-life in vivo [10], ZBP-ES was recommended to be taken on a daily basis in clinic.

PEGylation, the process of covalent attachment of polyethylene glycol (PEG) to the proteins or peptides, can overcome the short half-life problem. PEGylation masks the surface of the proteins and increases their molecular size, therefore protecting them from protease digestion and renal filtration, while at the same time increasing the protein’s stability and residence time in vivo [11]. The applicability and safety of this strategy have been well documented, and PEGylated proteins have been used in clinics for years. Our group and our partners have developed a second generation of ES by conjugation of a PEG molecule to the N terminus of wild-type ES, which is also known as M_2_ES, and have tested its safety and efficacy in preclinical and clinical studies [12, 13]. M_2_ES showed a broad spectrum of antitumor activities in several types of tumors (data to be published) and was well tolerated in rhesus monkeys [12]. Furthermore, in the phase I trial, M_2_ES was well tolerated in pancreatic adenocarcinoma patients when concurrently administered with gemcitabine [13]. Other groups have also generated mono-PEGylated ZBP-ES and examined its potency in animal models [14, 15].

The objective of this study was to comprehensively compare both the biophysical properties and biological activities of M_2_ES and mono-PEGylated ZBP-ES (MZBP-ES), and therefore provide a basis for better understanding the druggability and bioactivities of PEGylated ES variants.

## Methods

### Proteins, antibodies, and cell culture

The recombinant proteins used in this study were provided by Protgen Co., Ltd. (Beijing, China). ES and ZBP-ES were expressed in *E. coli*, refolded into native forms and purified. The N-terminal site-directed mono-PEGylation was conducted by addition of 20 mmol/L NaBH_3_CN and incubating the PEG2000 and proteins (PEG2000:protein = 1.5:1) at room temperature for more than 2 h, followed by purification.

We purchased primary antibodies against extracellular regulated protein kinases (Erk), phosphor-Erk, glyceraldehyde 3-phosphate dehydrogenase (GAPDH) (Santa Cruz Biotechnology, Santa Cruz, CA, USA), PEG (Abcam, Cambridge, UK), and turbo green fluorescent protein (tGFP) (OriGene, Rockville, MD, USA). Anti-ES antibody was from our lab stock.

Human microvascular endothelial cells (HMECs) were from our lab stock and cultured in Dulbecco’s Modified Eagle Medium (DMEM) containing 10% fetal bovine serum (Hyclone, Logan, UT, USA).

### Circular dichroism (CD)

The far-ultraviolet (UV) CD spectra were obtained by a Chirascan™-plus CD Spectrometer (Applied photophysics, Surrey, UK). ES or its variants were diluted in 5 mmol/L Tris–HCl, pH 7.4, to a final concentration of 10 μmol/L. For each protein, data were recorded three times and corrected by subtracting the baseline spectrum of the buffer.

### Tryptophan emission fluorescence

Tryptophan emission fluorescence is a good probe to monitor the subtle tertiary structural changes of ES [16]. Proteins (1 μmol/L) in Tris buffer were measured as previously described [17].

### Proteolysis assay

ES or its variants (0.5 mg/mL) were incubated with trypsin (0.2 mg/mL) in phosphate buffer saline (PBS) buffer (pH 7.4) at 37 °C [18]. At each indicated time point, reaction solutions were quickly removed and mixed with the sodium dodecyl sulfate–polyacrylamide gel electrophoresis (SDS–PAGE) loading buffer to stop the trypsin digestion reactions. The samples were subsequently subjected to SDS–PAGE and stained with the Coomassie dye.

### Guanidinium chloride (GdmCl)-induced unfolding

ES or its variants (0.8 μmol/L) were incubated in 5 mmol/L Tris–HCl, pH 7.4, containing GdmCl concentrations ranging from 0 to 6 mol/L. After incubation for 24 h at room temperature, GdmCl-induced denaturation was monitored by the tryptophan emission fluorescence intensity at 318 nm [5, 19]. Data were normalized by subtracting the baseline fluorescence intensity of the buffer. The linear extrapolation method was used to evaluate the value of the free change energy in the absence of the denaturant GdmCl ($\rm\Delta {\text{G}}^{\text{o}}_{{{\text{N}}{-}{\text{U}}}}$), the apparent slope of the plots (m), and the concentration of GdmCl at the midpoint of the unfolding transition (C_m_) as described by Santoro and Bolen [20].

### In vitro protein–protein interactions

The nucleolin-tGFP plasmid was transfected into HMECs. Nucleolin-tGFP proteins were immunoprecipitated following the procedure described in our previous study [21]. Subsequently, ES or its variants were incubated with the nucleolin-tGFP beads for 1 h at 4 °C. After washing with the lysis buffer (150 mmol/L Tris, 50 mmol/L sodium chloride, 1% NP40, protease inhibitor mixture, pH 7.4), the bead-bound proteins were subjected to Western blotting.

### Internalization assay

As previously described [22], HMECs were treated with 5 μg/mL ES or its variants for 1 h. Subsequently, cells were washed with acidic buffer (pH 3.5) and ice-cold PBS to remove cell surface-binding proteins. The amount of internalized proteins was examined by Western blotting or immunofluorescence.

### Western blotting

Protein samples were separated by SDS–PAGE and transferred to a polyvinylidene difluoride (PVDF) membrane. After blocking for 1 h at room temperature, the membrane was incubated with the indicated primary antibodies overnight and then to the corresponding horseradish peroxidase (HRP)-conjugated goat-anti-mouse or goat-anti-rabbit secondary antibodies for 1 h. The enhanced chemiluminescent substrate was added to the blot, and reactive bands were detected.

### Immunofluorescence

Cells were seeded directly on coverslips, fixed, and stained. After blocking with 5% goat serum, cells were incubated with the indicated primary antibodies and fluorescein isothiocyanate (FITC)-labeled secondary antibodies. The nucleus was stained with 4′,6-diamidino-2-phenylindole (DAPI). Images were captured using a Nikon A1 confocal microscope (Nikon Corporation, Tokyo, Japan).

### Transwell migration and wound healing

Transwell migration and wound healing assays were conducted as previously described [22]. Cells were treated with the indicated proteins for 6 and 48 h at 37 °C, respectively. All experiments were repeated twice.

### Statistical analyses

All data from the experiments were presented as means ± standard deviations (SDs). Differences between two groups were calculated by GraphPad (GraphPad Software, San Diego, CA, USA), and considered significant if the *P* value was <0.05 using a two-tailed Student’s *t* test.

## Results

### Structural analyses

**Fig. 1:**
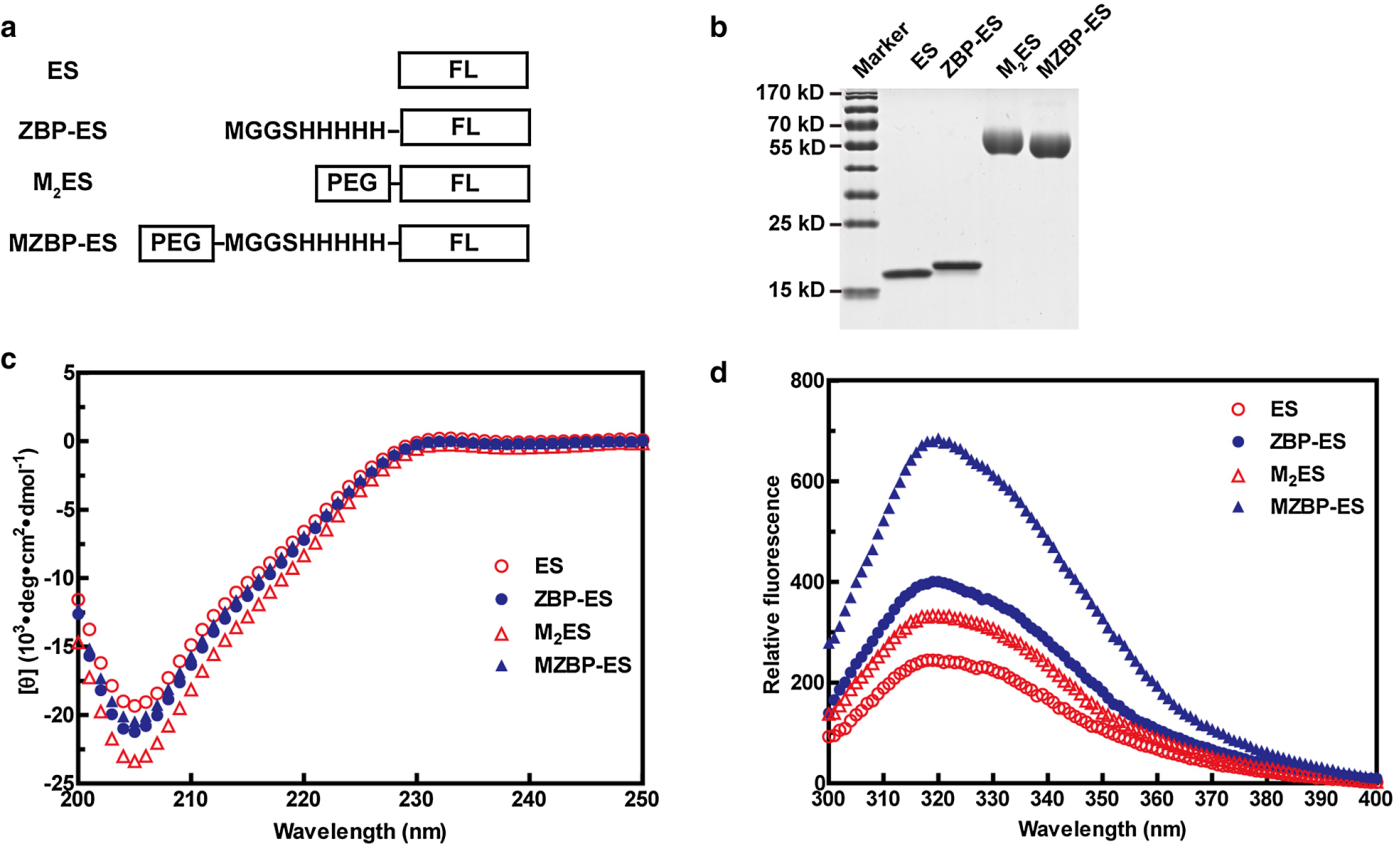
Sequence, purification, and structural analyses of endostatin (*ES*) variants. **a** Sequences of ES, zinc-binding protein-ES (*ZBP-ES*), mono-PEGylated ES (*M*_*2*_ *ES*), and mono-PEGylated ZBP-ES (*MZBP-ES*). *FL* full length. **b** The purity of ES and its variants examined by sodium dodecyl sulfate–polyacrylamide gel electrophoresis (*SDS*–*PAGE*). **c** Secondary structures of ES and its variants detected by far-ultraviolet circular dichroism (far-UV CD) spectra. **d** Tertiary structures of ES and its variants tested with tryptophan emission fluorescence

The schematic diagram shows the sequence of ES, ZBP-ES, M_2_ES, and MZBP-ES (Fig. 1a). The purity of these proteins is shown in Fig. 1b. The proteins were tested for secondary and tertiary structures using CD and tryptophan emission fluorescence. The CD spectra results revealed that no obvious change in the secondary structure was observed between ES and ZBP-ES, whereas slight differences were observed between M_2_ES and MZBP-ES (Fig. 1c). The maximal Trp fluorescence emission wavelengths (*λ*_max_) of all the ES variants were approximately 318 nm (Fig. 1d), indicating a relatively stable core structure of ES variants. However, the fluorescence intensities of ES and M_2_ES were higher than those of ZBP-ES and MZBP-ES, respectively, implying that the tertiary structure of ES is more compact than that of ZBP-ES and that the tertiary structure of M_2_ES is more compact than that of MZBP-ES.

Taken together, N-terminal modifications do not affect the core structure of ES, whereas the tertiary structure of M_2_ES is more compact than that of MZBP-ES.

### Protease digestion

**Fig. 2:**
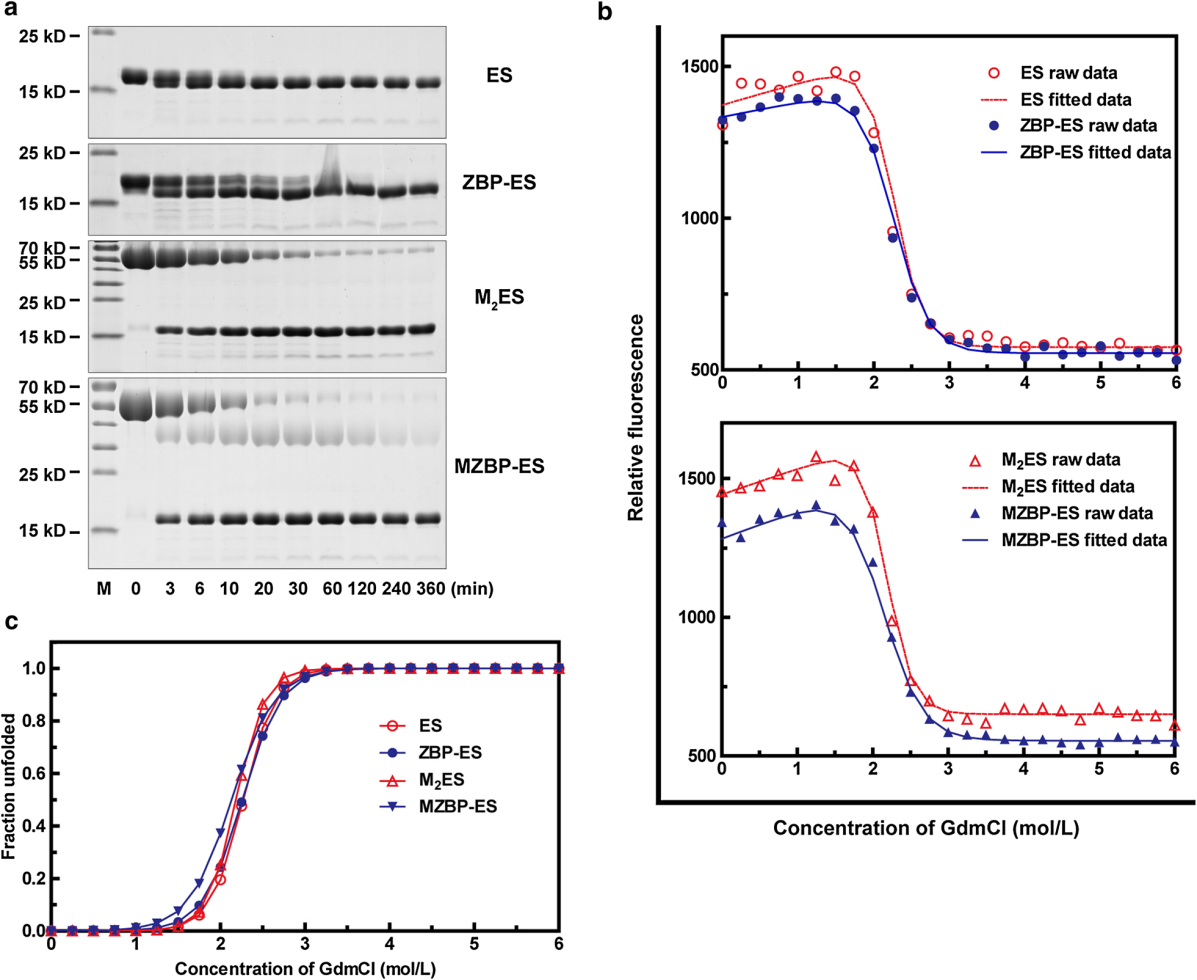
Stability of ES variants under certain conditions. **a** Trypsin digestion profiles of ES variants for the indicated time. **b** and **c** Proteins were incubated with different concentrations of guanidinium chloride (*GdmCl*), and GdmCl-induced denaturation of ES variants was monitored with tryptophan emission fluorescence intensity at 318 nm. **b** Raw data and fitted data of the unfolding process of ES, ZBP-ES (*upper panel*), and M_2_ES, MZBP-ES (*lower* *panel*). **c** Normalized data of the GdmCl-induced unfolding of ES variants

To identify the stability of ES variants, trypsin was incubated with ES variants for the indicated time periods. ES was quickly digested within 20 min, whereas ZBP-ES was not totally digested until 4 h (Fig. 2a). PEGylated proteins were overall more resistant to trypsin digestion compared with intact proteins. Interestingly, after incubation for 6 h, the residual amount of full-length M_2_ES was larger than that of MZBP-ES. Quantitative results showed that the initial reaction rate was 0.026 mg/(mL·min) for M_2_ES and 0.037 mg/(mL·min) for MZBP-ES. These results showed that M_2_ES was more resistant to trypsin digestion than MZBP-ES.

### GdmCl-induced unfolding

GdmCl-induced unfolding was used to detect the structural stability and unfolding cooperativity of ES variants. Slight changes were observed between the unfolding curves of ES and ZBP-ES (Fig. 2b, upper panel). These two proteins had a similar C_m_ value, whereas the modified ES exhibited lower values of ΔG_N–U_^o^ and m (Table 1). Moreover, the unfolding curve of M_2_ES had slight shifts towards higher GdmCl concentrations compared with that of MZBP-ES (Fig. 2b, lower panel), with increases in ΔG_N–U_^o^, m, and C_m_ (Table 1). These results showed that both the structural stability and unfolding cooperativity of M_2_ES were better than those of MZBP-ES (Fig. 2c).

**Table 1:** Parameters of the GdmCl-induced unfolding of ES variants

Protein	$\rm\Delta {\text{G}}^{\text{o}}_{{{\text{N}}{-}{\text{U}}}}$ (kJ/mol)	m (kJ·L/mol^2^)	C_m_ (mol/L)
ES	29.23	12.88	2.27
ZBP-ES	24.16	10.70	2.26
M_2_ES	31.27	14.31	2.18
MZBP-ES	20.57	9.65	2.13

*GdmCl* guanidinium chloride, $\varDelta G^{o}_{{N{-}U}}$ the free change energy in the absence of GdmCl, *m* the apparent m value defined by the linear extrapolation model, *C*_*m*_ the concentration of GdmCl at the midpoint of the unfolding transition, *ES* endostatin, *ZBP-ES* zinc-binding protein-ES, *M*_*2*_ *ES* mono-PEGylated ES, *MZBP-ES* mono-PEGylated ZBP-ES

### Biological activities

**Fig. 3:**
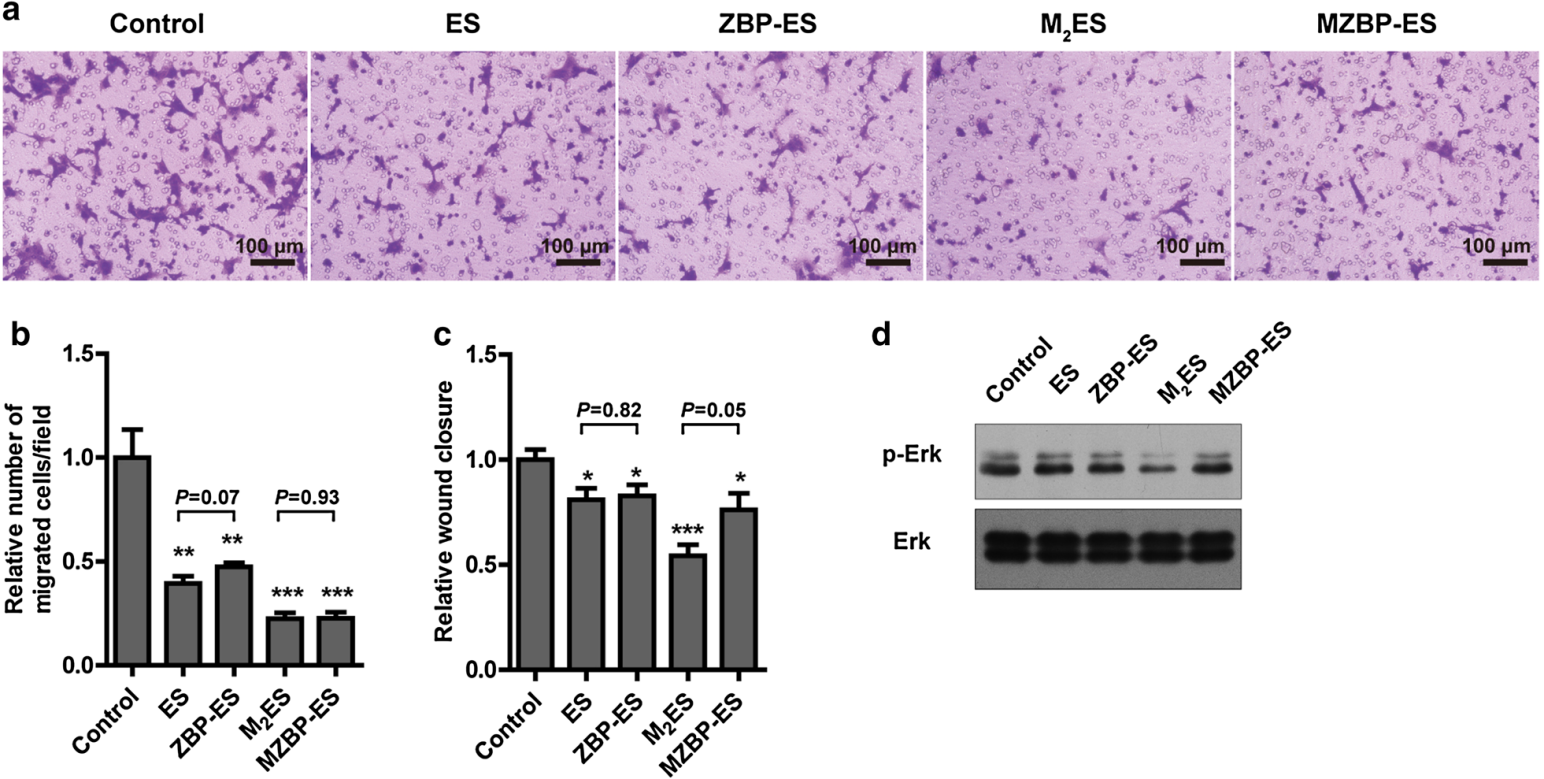
Biological activities of ES variants. **a** and **b** Representative images and quantified results of the Tranwell migration assay for endothelial cells. Human microvascular endothelial cells (*HMECs*) were seeded into the upper well of the Boyden chamber and treated with the indicated proteins for 6 h. Cells were stained with crystal violet, photographed, and counted. **c** In the wound healing assay, the HMEC monolayer was scratched and cultured in serum-free media in the absence or presence of the indicated proteins for 48 h. Cells were photographed at 0 and 48 h, respectively, and the relative wound closure was quantified. **d** Erk phosphorylation of ES variant treatments examined by Western blotting. *Error bars* indicate standard deviations (SDs). *P< 0.05, **P< 0.01, ***P< 0.001, vs. control

We next investigated the effects of ES variants on endothelial cell activities. ES and its variants significantly reduced endothelial cell migration, and PEGylated proteins were more efficient compared with intact proteins (Fig. 3a, b). In the Transwell assay, M_2_ES and MZBP-ES inhibited endothelial cell migration to an equivalent extent. The wound healing scratch results showed that M_2_ES was slightly more potent than MZBP-ES in retarding wound healing (Fig. 3c).

ES was shown to bind to the vascular endothelial growth factor receptor 2 (VEGFR2) on the surface of endothelial cells and disrupt vascular endothelial growth factor (VEGF)-induced activation of mitogen-activated protein kinase/extracellular regulated protein kinases (MAPK/Erk) [23]. All ES variants inhibited Erk phosphorylation (Fig. 3d). ZBP-ES was more potent in reducing Erk activation compared with ES, whereas the blockage effect of M_2_ES was more potent than that of MZBP-ES.

In summary, M_2_ES and MZBP-ES had comparable effects on the inhibition of cell migration, whereas M_2_ES was more efficient in disrupting Erk activation.

### Internalization by endothelial cells and interactions with cell surface receptors

**Fig. 4:**
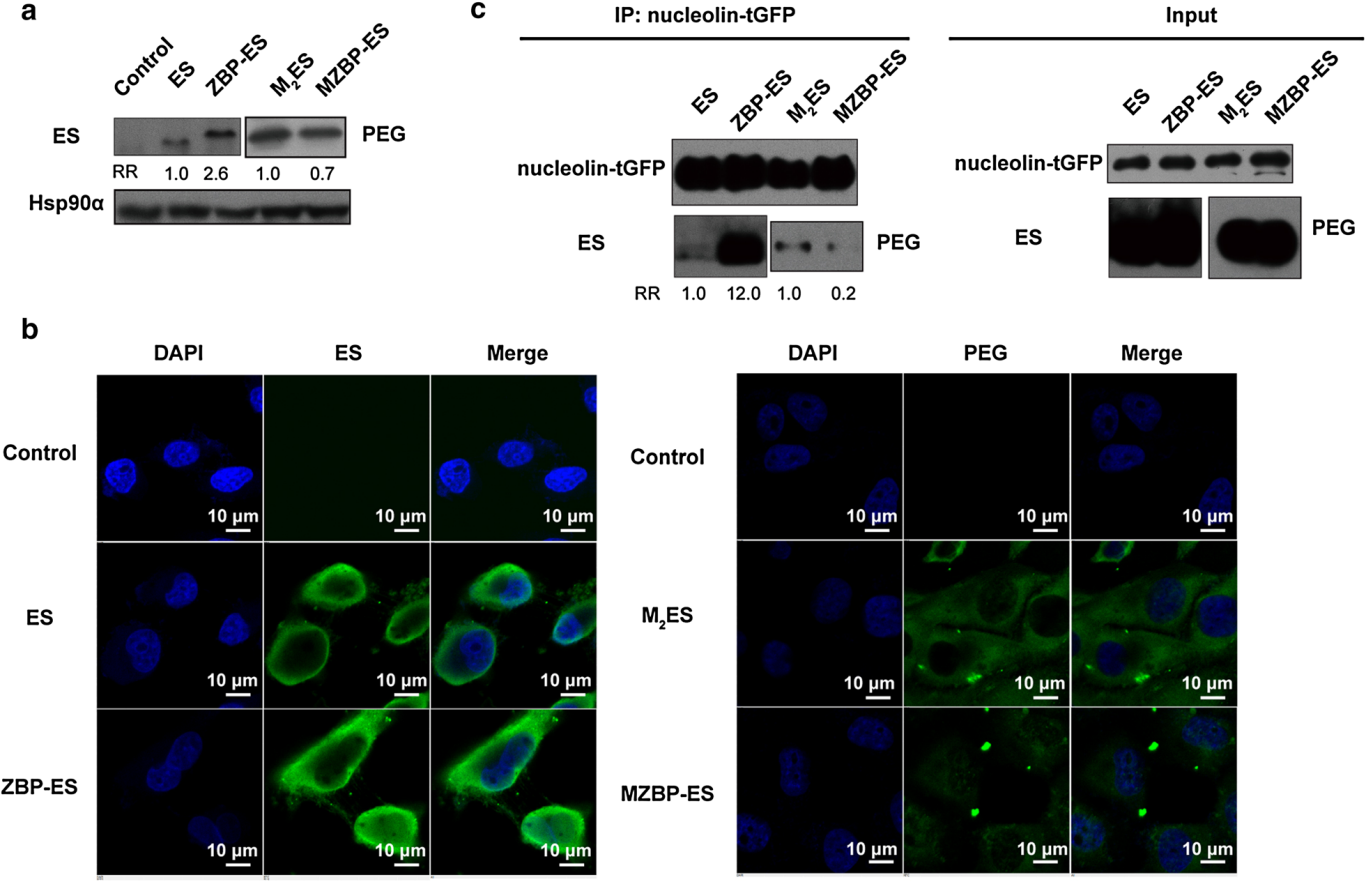
Cellular uptake and interaction of ES variants with nucleolin. **a** ES variant internalization by endothelial cells determined by Western blotting. HMECs were treated with the indicated proteins, washed to remove cell surface-bound proteins, and used for Western blotting with anti-ES or anti-PEG antibodies to analyze the amount of internalized proteins. Heat shock protein 90α (Hsp90α) was used as the loading control. **b** Internalization of ES variants into endothelial cells detected by immunofluorescence. DAPI, 4′,6-diamidino-2-phenylindole. **c** Interaction of ES variants with nucleolin-turbo green fluorescent protein (nucleolin-*tGFP*) in vitro. Nucleolin-tGFP proteins were expressed in endothelial cells, and immunoprecipitated with anti-tGFP antibody and Protein G beads. The purified nucleolin-tGFP beads were incubated with 0.5 μg indicated ES variant at 4 °C for 1 h. Then, the beads were washed and collected, and Western blotting was used to detect the amount of bead-bound proteins. *RR*, relative ratio

Internalization is important, if not critical, for the biological functions of ES [24]. Increasing the uptake of ES via the addition of cholesterol chelating agents [22] or engineering a peptide to its N terminus [25] dramatically enhanced the therapeutic efficacy of ES. We determined the uptake of ES and its variants by HMECs and found that ZBP-ES was more efficient in entering the cells than ES (Fig. 4a), which validates the data from our previous study [16]. Intriguingly, more M_2_ES proteins accumulated in the cells than MZBP-ES (Fig. 4a). Consistent results were obtained by immunofluorescence (Fig. 4b).

Nucleolin is a functional receptor of ES and participates in the ES internalization process [26]; therefore, we explored whether the distinction of ES variant internalization was attributed to different binding affinities to nucleolin. Consistent with the internalization results (Fig. 4a, b), ES showed weaker interaction with nucleolin than ZBP-ES, whereas M_2_ES showed increased interaction with nucleolin compared with MZBP-ES (Fig. 4c).

Collectively, M_2_ES exhibited an increased cellular uptake than MZBP-ES, possibly caused by the enhanced interaction with its functional receptor nucleolin.

## Discussion

In this study, we demonstrate that M_2_ES not only has a more compact tertiary structure than MZBP-ES but also exhibits more resistance to trypsin digestion and GdmCl-induced unfolding. Although M_2_ES shows comparable inhibitory effects on endothelial cell migration and wound healing compared with MZBP-ES, M_2_ES is more efficient in entering the cells than MZBP-ES, possibly due to enhanced interaction with its functional receptor nucleolin.

M_2_ES is more stable towards trypsin digestion than MZBP-ES (Fig. 2a). Our previous study revealed that the endostatin digestion process contains two stages: cleavage of the first four residues (HSHR) during the first stage and further digestion of the dominant product in the second stage [18]. We therefore supposed that the PEG molecule in M_2_ES masks the HSHR residues and therefore protects it from degradation by trypsin, whereas the PEG molecule of MZBP-ES covers the MGGSHHHHH part, instead of the first four residues, making MZBP-ES more susceptible to protease digestion.

Endostatin is well established as an antiangiogenic factor; therefore, our study focused on the direct impacts of ES and PEGylated ES on endothelial cells. However, endothelial cells are not the only target of ES. Our recent study showed that adipocytes were also targeted by ES. ES can inhibit adipogenesis and dietary-induced obesity and its related metabolic disorders, including glucose intolerance, insulin resistance, and hepatic steatosis [27]. Although both intensive and extensive researches were performed on ES, and it appeared that there was not much progress on this protein, this new discovery suggests a potential expanded application of ES for the treatment of obesity. Moreover, this discovery opens new avenues for this protein and indicates that this is only the beginning.

## Conclusions

Collectively, these findings suggest that M_2_ES is a more suitable drug candidate in comparison with MZBP-ES and provides a basis for further endostatin-based drug development.
